# Microtubule-dependent balanced cell contraction and luminal-matrix modification accelerate epithelial tube fusion

**DOI:** 10.1038/ncomms11141

**Published:** 2016-04-12

**Authors:** Kagayaki Kato, Bo Dong, Housei Wada, Miho Tanaka-Matakatsu, Yoshimasa Yagi, Shigeo Hayashi

**Affiliations:** 1 Laboratory for Morphogenetic Signaling, RIKEN Center for Developmental Biology, 2-2-3 Minatojima-minamimachi, Chuo-ku, Kobe, Hyogo 650-0047, Japan; 2 Feinberg School of Medicine, Northwestern University, 303 East Chicago Avenue, Chicago, Illinois 60611, USA; 3 Division of Biological Science, Graduate School of Science, Nagoya University, Furo-cho, Chikusa-ku, Nagoya 464-8602, Japan; 4 Department of Biology, Kobe University Graduate School of Science, 1-1 Rokko-dai, Nada-ku, Kobe, Hyogo 657-8501, Japan

## Abstract

Connection of tubules into larger networks is the key process for the development of circulatory systems. In *Drosophila* development, tip cells of the tracheal system lead the migration of each branch and connect tubules by adhering to each other and simultaneously changing into a torus-shape. We show that as adhesion sites form between fusion cells, myosin and microtubules form polarized bundles that connect the new adhesion site to the cells' microtubule-organizing centres, and that E-cadherin and retrograde recycling endosomes are preferentially deposited at the new adhesion site. We demonstrate that microtubules help balancing tip cell contraction, which is driven by myosin, and is required for adhesion and tube fusion. We also show that retrograde recycling and directed secretion of a specific matrix protein into the fusion-cell interface promote fusion. We propose that microtubule bundles connecting these cell–cell interfaces coordinate cell contractility and apical secretion to facilitate tube fusion.

## Abstract (web summary)

During tracheal tube fusion in *Drosophila*, a pair of tip cells form an adherens junction and then fuse their plasma membranes. Here the authors show that a balanced pulling force mediated by myosin and microtubules, as well as localized deposition of matrix, promotes plasma membrane fusion.

## Graphical abstract

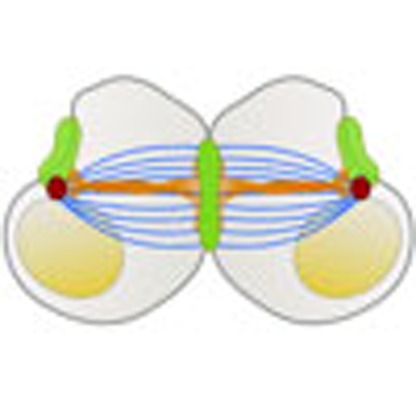

Organ formation and modifications in tissue architecture occurring during development involve deconstructing both cell adhesion and the extracellular matrix^1^,^2^,^3^, thereby converting cells into a migratory state with a new tissue identity; this is known as epithelial–mesenchymal transition^4^,^5^. Although epithelial cells and mesenchymal cells have contrasting properties, the molecular mechanisms behind these properties are not entirely mutually exclusive. Some epithelial cells acquire the ability to migrate while retaining cell–cell adhesion, allowing these cells to migrate as a group^6^,^7^,^8^,^9^. This is possible due to the polarized nature of the epithelia; the apical side retains the cell–cell interface, while migratory protrusions form on the basal cell surface. On reaching their destination, these migratory epithelia are integrated into the target tissues. However, how migratory epithelia find their target tissues, establish new cell–cell interfaces and integrate with the target tissue are poorly understood.

Studies of the formation of adhesive interfaces in cultured cells showed that contact with actin-enriched filopodia initiates the assembly of the adherens junction^10^,^11^ and suggested that microtubules are involved in this process^12^. As the formation of cell junctions involves coordinating the assembly of the cell adhesion complex and the generation of tension in each side, the cells forming the junction must be observed simultaneously to understand how mechanical coupling is controlled at the tissue level.

Here, to examine how cell adhesion and cell contractility are coupled to establish a new cell interface, we examined the anastomosis formation in the *Drosophila* tracheal system. In this process, a pair of tip cell (hereafter called fusion cell, FC) forms an adherens junction *de novo* (Fig. 1a,b) and deposit extracellular matrix materials into newly forming lumen. Each FC is converted into a torus shape by plasma membrane fusion via the inner leaflet^16^,^17^,^18^, under the control of endosome and microtubule-associated protein Arl3 (refs 8, 19, 20). Interestingly, plasma membrane fusion always occurs simultaneously in each FC, suggesting cell shape change and membrane trafficking in each FC pair is highly coordinated. However, how cell adhesion, cell-shape change and membrane trafficking are regulated coordinately in pairs of FCs is poorly understood.

We show that FCs form a *de novo* adherens junction at the contact site through stabilization by a mechanism requiring actomyosin and microtubules; this mechanism applies a balanced pulling force to flatten the FCs. In addition to this cell-intrinsic contractile force, the preferential deposition of molecules via the apical secretion pathway promotes maturation of the matrix in the lumen that forms in the FC contact interface and helps to fuse the plasma membranes.

## Results

### F-actin and microtubule dynamics in migrating FCs

**Figure 1:**
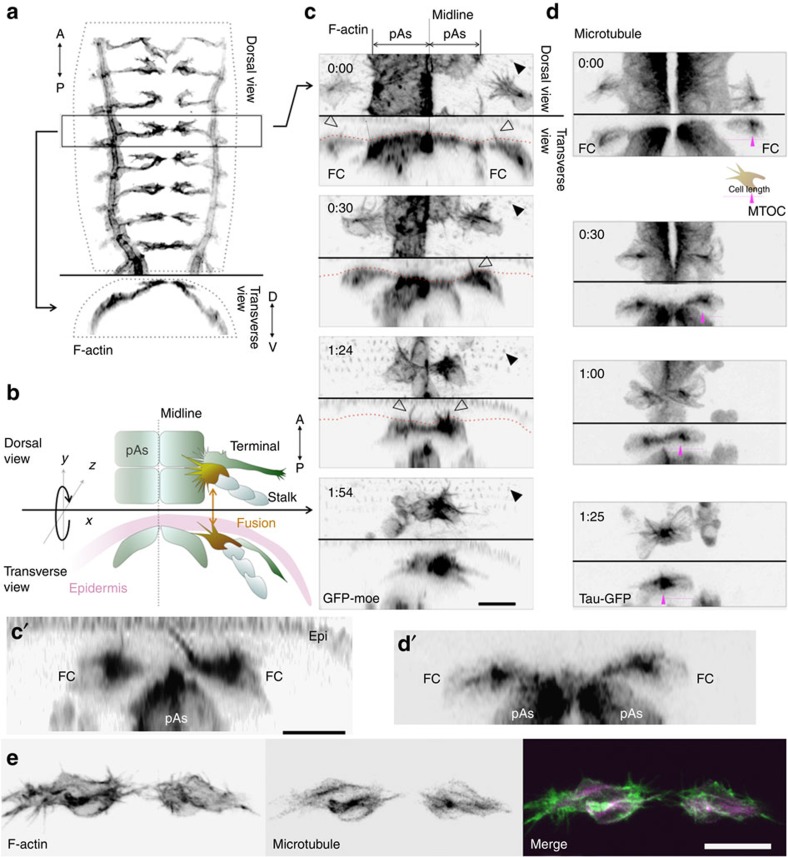
Dorsal-branch migration and fusion of the *Drosophila* trachea. (**a**) A stack of confocal GFP images shows the tracheal system of a stage-15 embryo carrying *btl>GFP-moe*. (**b**) Spatial relationship of the migrating tracheal branch and the epidermis; FCs, terminal cells and stalk cells of the dorsal branch and peripheral amnioserosa (pAs) are indicated. (**c**) Time course of dorsal-branch migration and fusion, shown by F-actin labelling with *esg_FC-Gal4* and *UAS-GFP-moe* (FC and pAs), and with sqh-GFP-moe (epidermis). Each panel shows dorsal (top half) and transverse views (bottom half) of images at the indicated time points (top left of the image). Filled triangles indicate F-actin-rich denticles of the apical surface of the epidermis; open triangles indicate FC filopodia penetrating vertically into the epidermis and nearly reaching the apical surface. Red dotted line indicates the basal epidermal surface. (**c'**) Enlarged view of the vertical filopodia (1:30). (**d**) Microtubule distribution during tracheal-branch fusion, revealed by expressing the tau-GFP marker in FCs and pAs by the *esg_FC-Gal4* driver. Magenta triangles indicate MTOCs. (**d'**) Enlargement of **d**, 0:30 time point. (**e**) Double labelling of microtubules (GFP-tau) and F-actin (GFP-moe). Scale bar, 10 μm.

FCs expressing the F-actin markers GFP-moesin (Fig. 1c; see also Supplementary Movie 1) or lifeact-GFP (Supplementary Fig. 1a), each driven by an FC-specific enhancer (*esg_FC* enhancer; see Methods), were closely associated with the basal surface of the dorsal epidermis and showed numerous F-actin-enriched filopodia extending towards the dorsal midline^18^,^21^. We also observed invasive filopodia that extended vertically from these FCs into the epidermal layer (Fig. 1c open triangles, Fig. 1c′ and see also Supplementary Fig. 1a); their location did not correspond to epidermal cell junctions, suggesting that the filopodia penetrated the epidermal cells. While migrating, the FCs appeared flat with numerous forward-extending filopodia (Fig. 1c, time 0:00 h), but on reaching their target, their shape became more compact, with the lumen extending from the side of the stalk (Fig. 1c, time 1:54 h). Microtubule labelling with GFP-tau revealed extensive microtubule arrays emanating from the microtubule-organizing centre (MTOC) at the proximal side of the cell (Fig. 1d, time 0:00 h; see also Supplementary Fig. 1b,c and Supplementary Movie 2) and F-actin labelling revealed many, F-actin-rich protrusions extending forward to reach the target cell (Fig. 1e and see also Supplementary Movie 3). On FC contact with its partner FC, the MTOC moved to the contact site (Fig. 1d, time 1:25 h). The vertical protrusions revealed by F-actin marking were not observable with microtubule labelling (compare Fig. 1c′ and Supplementary Fig. 1b for another microtubule marker GFP-Jupiter).

### A polarized flux of E-Cadherin to the new cell junction

**Figure 2:**
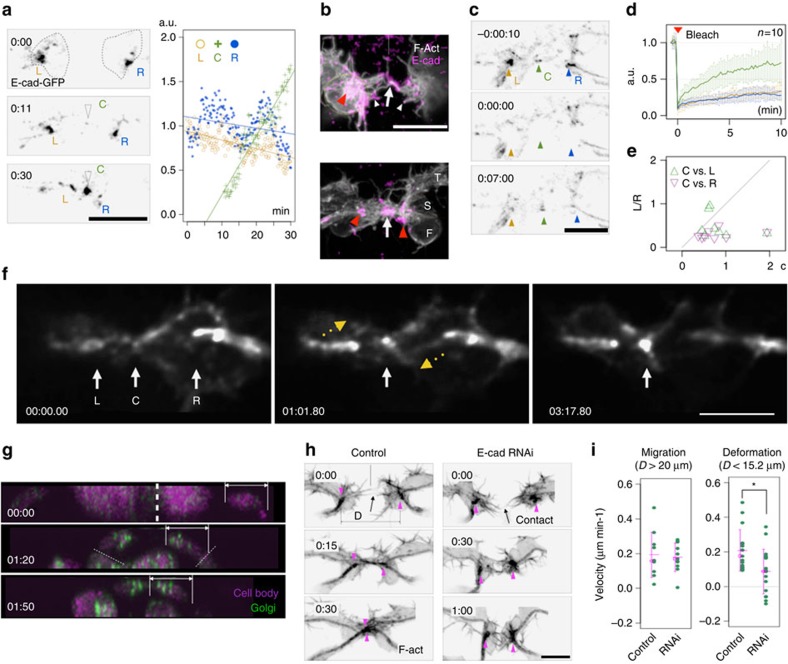
E-cadherin accumulation and trafficking. (**a**) E-cadherin accumulation during formation of the cell junction (C) between FCs is shown by time-lapse movie (left) and by quantification of the accumulated E-cadherin-GFP (right). L and R, left and right FC–stalk boundaries. (**b**) E-cadherin accumulated at two sites in FC filopodia: in FCs making contact (arrow) and at the tip of forward-extending filopodia (white arrowheads). Red arrowhead, FC–stalk boundary; S, stalk cell; T, terminal cell. FRAP analysis (**c**) and quantification (**d**) of E-cadherin-GFP accumulation. Error bar indicate s.d. (**e**) A scatter plot of C versus L/R signals (Fc) after recovery showing the preferential addition of E-cadherin-GFP to the newly forming junction between FCs. (**f**) Intracellular E-cadherin-GFP trafficking: the GFP signal accumulated progressively in the FC contact site (C, white arrow) over a period of ∼3 min. Yellow arrows in the central panel show cytoplasmic E-cadherin-GFP streaming towards the plasma membrane. (**g**) Relocalization of the Golgi apparatus to the front end of FCs during new junction formation, visualized by imaging Grasp65-GFP driven by *esg_FC_Gal4*. (**h**) RNAi inhibition of *de novo* E-cadherin synthesis interfered with branch fusion. (**i**) Velocity measurements demonstrated that branch migration before branch contact was normal, but the cell deformation after contact was significantly delayed in the E-cadherin RNAi embryo (*Student's *t*-test, *P*<0.05). Scale bar, 10 μm.

When two FCs came within a proximity of 15.2 μm (on average), fluorescent markers for E-cadherin (Fig. 2a)^22^ and its associated protein p120ctn (Supplementary Fig. 3a)^23^, accumulated at the contact site. Immunostaining revealed endogenous E-cadherin localized to the extending edge of forward-reaching filopodia on the tip cells (Fig. 2b top). This punctate E-cadherin localization was not observed on the filopodia of terminal cells or stalk cells (Fig. 2b bottom). To monitor the dynamics of E-cadherin transport, we imaged E-cadherin-GFP fluorescence during the fusion process. We found that the E-cadherin-GFP level remained fairly constant at the junction between the FC and stalk cell (Fig. 2a, position and plot marked with ‘L' and ‘R'), but increased steadily at the FC interface, surpassing the intensity at the FC–stalk cell interface within 20 min after contact (Fig. 2a). We next monitored the E-cadherin-GFP turnover rate at each cell interface by fluorescence recovery after photobleaching (FRAP) analysis. All three E-cadherin-GFP foci (L, R and C) were photobleached simultaneously and their rate of fluorescence recovery was measured (Fig. 2c arrowheads). The fluorescence recovery rate and the mobile E-cadherin-GFP fraction were significantly higher at the FC interface than at the L and R foci (Fig. 2d,e). Imaging with high spatiotemporal resolution revealed that E-cadherin-GFP appeared on the free surface of the plasma membrane, with occasional streams from the cytoplasm to the membrane surface, and then accumulated at the FC contact site (Fig. 2e and Supplementary Movie 4). In addition, Golgi apparatus in FCs became progressively enriched near the contact site (Fig. 2g).

The low E-cadherin-GFP turnover at the FC–stalk cell interface suggests that the exchange rate of the E-cadherin complex at this junction, which was already present at the time of dorsal branch (DB) migration, is low. To analyse E-cadherin's contribution, we reduced its synthesis by double-stranded RNA-mediated gene knockdown (Supplementary Fig. 3b). Expressing E-cadherin double-stranded RNA in tracheal cells with the trachea-specific *btl-Gal4* driver reduced the level of E-cadherin in DB stalk cell junction by ∼27% (Methods), but did not compromise the overall tracheal morphology or the migration speed of the DB before the FCs came into contact with each other (Fig. 2h,i), suggesting that the amount of E-cadherin gene product in this experimental condition was sufficient to sustain the tracheal tissue architecture. However, the FC contraction was significantly delayed after contact and the interface between the FCs failed to mature (Fig. 2h,i). These results suggest that newly synthesized E-cadherin is preferentially partitioned to the distal side, to form the new cell interface.

### FC contraction by a myosin-dependent pulling force

**Figure 3:**
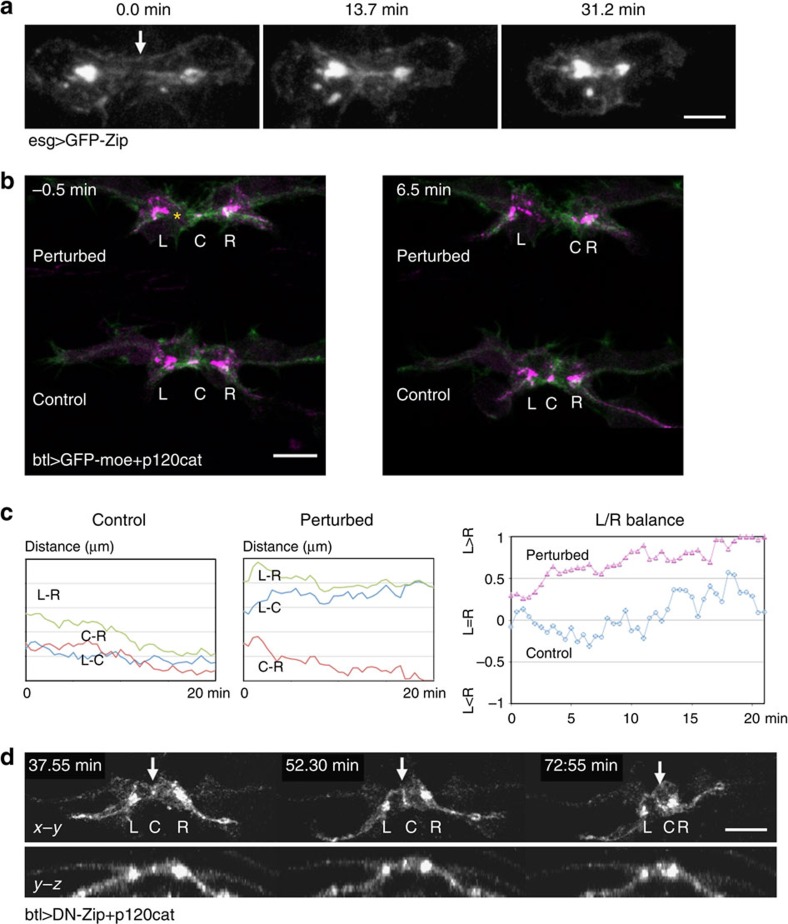
FC contraction. (**a**) GFP-myosin heavy chain formed a longitudinal track connecting two adherens junctions in an FC. (**b**) Laser perturbation of FC contraction: the left-side cell (asterisk) in a new FC pair was illuminated by infrared laser at the time of contact. (**c**) The location of each cell-adhesion site (L, C and R) was tracked and the distance between sites was plotted. Laser perturbation caused significant deviations in L/R balance values (*N*=3, a representative case is shown). (**d**) Fusion was inhibited by a dominant-negative form of myosin. White arrow indicates the FC contact site. Scale bar, 10 μm.

We next examined the role of myosin II in the contractile force within the FC. When expressed in FCs, a green fluorescent protein (GFP) fusion protein of the myosin heavy chain Zipper formed longitudinal bundles at the time of FC–FC contact, to connect the adherens junctions on each FC (Fig. 3a and Supplementary Movie 5). These myosin bundles became shorter as the FCs contracted and became more compact (Fig. 3a). Similar myosin bundles were observed in the dorsal-trunk FCs (Supplementary Fig. 4a).

To determine whether FC contraction is driven by a cell-autonomous force, we assessed the contractile state of laser-perturbed FCs. A microwave-length infrared laser (1,440 nm) effectively penetrated the epidermal layer and produced sufficient heat at the focal point to induce a heat-shock response^24^,^25^. Infrared laser illumination targeted to one of the paired FCs caused a cell-specific relaxation of cell contractility (Fig. 3b), with a simultaneous excessive contraction in the intact FC. The progression of the fusion process, assessed by shortening of the L–R length, was also arrested (Fig. 3c and Supplementary Movie 6, *N*=3). This result supports the idea that FC pairs normally exert nearly equal pull on each other to maintain their symmetric appearance, and that this balanced pulling force is required to pull FCs closer together. Furthermore, expressing a dominant-negative form of the myosin heavy chain interfered with FC contraction (Fig. 3d and Supplementary Movie 7, 100% *N*=9, 3 embryos) and fusion (Supplementary Fig. 4e,f).

### Polarized microtubule organization in the FC

**Figure 4:**
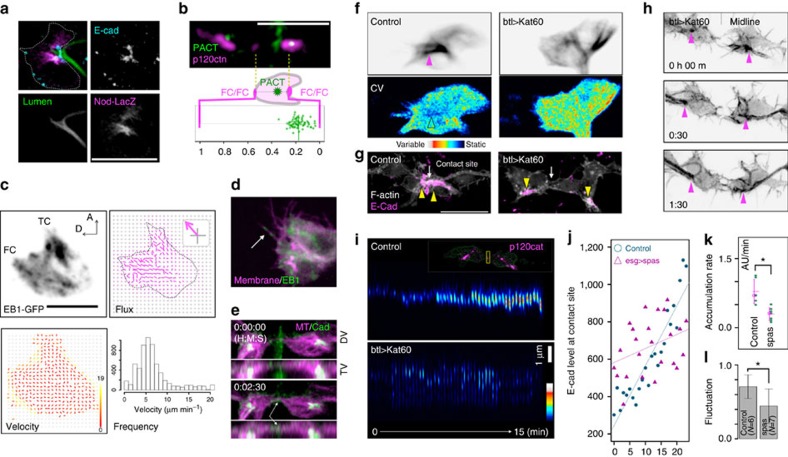
Microtubule dynamics in FCs. (**a**) The polarization of FC microtubules during migration. The FC is outlined with a dotted line. The microtubule minus-end marker Nod-LacZ was concentrated at the FC–stalk cell boundary, which was marked with E-cadherin and lumen (chitin) staining. (**b**) The centriole marker GFP-PACT localized near the FC–stalk cell boundary; the lower panel shows the relative GFP-PACT localization along the axis connecting the FC–FC and FC–stalk cell boundaries during fusion. (**c**) Localization of the microtubule plus-end marker EB1-GFP and the flux and velocity distribution deduced by particle image velocimetry (PIV) analysis. (**d**) Multiple EB1-GFP comets entered a single filopodia. (**e**) E-cadherin-GFP accumulated on microtubule tracks at the FC contact site. (**f**) Kat60 destabilized the microtubule lattice in FCs. The distribution of tau-RFP (top, averaged for 20 frames of 10-s intervals) and its pixel-wise coefficient of variation in a 100-s period (bottom, averaged as in the top panel) are shown. (**g**) Kat60 inhibited E-cadherin accumulation at the FC contact site. (**h**) Kat60 inhibited tracheal-branch fusion. FCs contacted each other using filopodia, but did not form a stable adhesion interface. (**i**) Time course of p120ctn-RFP accumulation at the FC contact site. The region of interest (ROI) was set at the FC contact site (yellow box in inset); its intensity profile over a 15-min period is shown. (**j**) Time course of E-cadherin-GFP accumulation (arbitrary unit) in control and *esg_FC-Gal4>Spas* FCs. (**k**) The rate of E-cadherin-GFP accumulation. (**l**) Fluctuation of E-cadherin-GFP accumulation (*R*^2^ value). Error bar indicates s.d. Scale bar, 10 μm.

We next studied the detailed organization of microtubules. The microtubule minus-end marker Nod-LacZ (ref. 26) and the centriole marker GFP-PACT (ref. 27) were concentrated near the FC–stalk cell interface (Fig. 4a,b). From this proximal MOTC, microtubules extended in a fan-shaped pattern to the migrating FC's leading edge (Fig. 1d and Supplementary Fig. 1c). We also imaged the microtubule plus-end marker EB1-GFP in FCs (Fig. 4c and Supplementary Movie 8); particle image velocimetry analysis revealed rapid, comet-like EB1-GFP signals with a mode velocity of 5 μm min^−1^ moving towards the FC's leading edge (Fig. 4c) and an EB1-GFP population moving at even higher velocities (>10 μm min^−1^) in the periphery (Fig. 4c). Dual-colour imaging of EB1-GFP and the cell membrane revealed that the microtubule plus ends projected to the cortical region of the leading edge and extended repeatedly into the filopodial protrusions (Fig. 4d and Supplementary Movie 9). The microtubule-filled protrusions were found most frequently in the leading edge, which made contact with the partner FC at the midline and accumulated E-cadherin-GFP (Fig. 4e and Supplementary Movie 10).

### Requirement of microtubules for tracheal fusion

To reveal the role of polarized microtubule arrays in tracheal-tube fusion, we perturbed the FC microtubule organization by expressing Katanin p60 (Kat60), a catalytic subunit of the microtubule-severing factor Kat60/p80 (refs 28, 29, 30). Microtubules in control FCs, visualized by the marker GFP-tau, formed dense central bundles directed towards the leading edge, with diffuse fractions filling the rest of the cell (Fig. 4f top and Supplementary Movie 11). Analysis of the signal variance in a 200-s interval (20 frames) revealed stable central microtubule bundles and an unstable diffuse fraction (Fig. 4f bottom). In FCs expressing Kat60, the microtubule bundle was misdirected and became unstable (high signal variance, Fig. 4f), indicating that the ectopic Kat60 could destabilize the microtubules. In this condition, endogenous Zip cables connecting the adherence junctions of FCs was reduced (Supplementary Fig. 2). DBs expressing Kat60 migrated towards the dorsal midline and maintained normal-shaped stalk cells and the FCs contacted each other using filopodia. However, the fusion process was arrested, because the filopodia contact site failed to accumulate E-cadherin or mature into a tight cell contact^18^ (Fig. 4g,h). In control trachea, signals from the adherens junction marker p120ctn-mRFP increased steadily in the contact site. However, in cells expressing Kat60, the p120ctn-mRFP accumulation was unstable, showing a ‘blinking' pattern (Fig. 4I and see also Supplementary Movie 12). These results indicate that microtubule destabilization inhibited the accumulation of the E-cadherin complex during fusion^18^.

To further elucidate the role of microtubules in forming the adherens junctions, we expressed Spastin (Spas), another microtubule-severing factor^31^, in FCs. Spas was previously demonstrated to inhibit tracheal branch migration and fusion^15^,^18^. We found Spas to be less disruptive to the fusion process than Kat60 (Methods), making it suitable for quantifying the E-cadherin-GFP accumulation at the contact site over time. The rate of E-cadherin-GFP accumulation was estimated by the slope of a fitted line (Fig. 4j,k) and the coefficient of determination (*R*^2^-value) was used as a measure of fluctuation (Fig. 4l). Spas reduced the E-cadherin-GFP accumulation rate and increased the fluctuation (lower *R*^2^-value; Fig. 4l). Despite these defects, FCs expressing Spas eventually accumulated E-cadherin-GFP and completed branch fusion. Taken together, these results indicate that proper microtubule organization is required for stable, persistent E-cadherin accumulation to the new cell-adhesion sites in tracheal-branch fusion.

### Microtubules coordinate deformation of paired FCs

**Figure 5:**
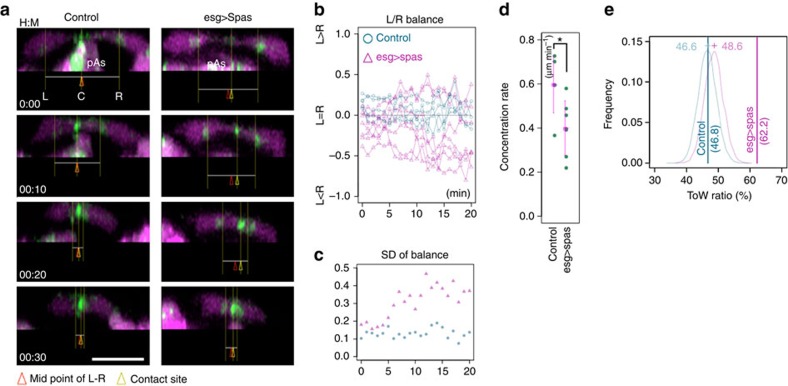
The balanced contraction of paired FCs depends on microtubules. (**a**) Microtubule inhibition disrupted the balanced contraction: *x*–*z* views show FC pairs in control and Spas-expressing embryos (esg_FC-Gal4). Green, E-cadherin-GFP; magenta, tagRFP. Arrowheads and lines indicate cell-adhesion sites. (**b**) Plot of the L/R balance values. Spas expression caused greater deviations from a balanced condition, which was represented by a value of 0. The onset of E-cadherin-GFP accumulation at the FC–FC interface was set as 0 min in the time series of each embryo. (**c**) Fluctuations in contraction: the s.d. of the L/R balance value was plotted against time. (**d**) A contraction rate plot based on the L–R length decrease (*Student's *t*-test, *P*<0.05). (**e**) Evaluation of ToW conditions: vertical bars indicate the frequency of contraction/relaxation in FC pairs for each time interval measured in the two genotypes. Curves indicate the simulated distribution of numerically calculated frequencies of paired contraction/retraction events, assuming that contraction and retraction occur randomly in each FC (10,000 trials). The value calculated for control FCs was slightly smaller than that for Spas-expressing FCs due to the difference in contraction rate (**d**). The ToW value for Spas-expressing FCs (62.2%) was far greater than the value expected from the random hypothesis, suggesting that uncoordinated contraction/retraction events occurred more frequently in the FCs expressing Spas. Scale bar, 10 μm.

We next analysed the dynamics of cell-shape change during the fusion process by monitoring the distance between the FC contact site (C) and the FC–stalk cell boundaries at the left (L) and right (R) side branch. In control embryos, FC pairs maintained their symmetry, with nearly equal C–L and C–R lengths (Fig. 5a–c and Supplementary Movie 13). However, FCs that expressed Spas deformed more slowly, as measured by the speed at which the L–R length decreased (Fig. 5d), and failed to maintain a balanced bilateral cell contraction (Fig. 5a) as measured by the L/R balance; the L/R balance was calculated as (*a*−*b*)/(*a*+*b*), where *a*=C–L and *b*=C–R (Fig. 5b). The degree of fluctuation, based on the s.d. of the L/R balance, remained low in control cells. However, imbalances in cell length began appearing in *esg_FC>Spas* cells 5 min after the first contact between FC cells (Fig. 5c). These findings indicated that proper microtubule organization is required for balanced cell contraction.

We further analysed the mechanism of the balanced cell contraction. We hypothesized that asymmetry in the length of paired FCs is caused by an imbalance in contractile forces between the cells, creating a back-and-forth tug-of-war (ToW) situation. To demonstrate this concept, we calculated the contraction speed of each FC at every time interval after contact. We used the contraction speed of viscoelastic cell materials, which is proportional to the contractile force^32^, as a surrogate of cellular force. Negative and positive values indicate contractile and relaxed state of each FC, respectively. If both FCs contracted or relaxed simultaneously at a given time point after contact, the contractile force was considered balanced. If one cell contracted while the other relaxed, the event was considered imbalanced and in the ToW state. We pooled a number of such cell-shape change events and calculated the percentage of ToW imbalances (Fig. 5e; ToW ratio, solid vertical bar). The ToW ratio was higher in the *esg_FC>Spas*-expressing cells (62.2%, 7 time series) than in control cells (46.8%, 4 time series). To determine whether this deviation might be caused by random drift, we ran a simulation to calculate the ToW ratio; this simulation assumed that FCs contracted independently with the deformation rate in normal distribution (Fig. 5e; mean displacement, −0.22±0.65 μm min^−1^ for control and −0.17±0.79 μm min^−1^ for *esg_FC>Spas* cells; see Methods). The calculated probability distributions of control and *esg_FC>Spas* conditions were similar to the control value (Fig. 5e, thin curves, 10^4^ trials each), but deviated greatly from the measured *esg_FC>Spas* value. Therefore, the ToW ratio deviation observed in *esg_FC>Spas* cell pairs was due to an imbalance of forces in the paired FCs, leading to ToW-type contractions.

### Directed apical Serp secretion to the FC interface

**Figure 6:**
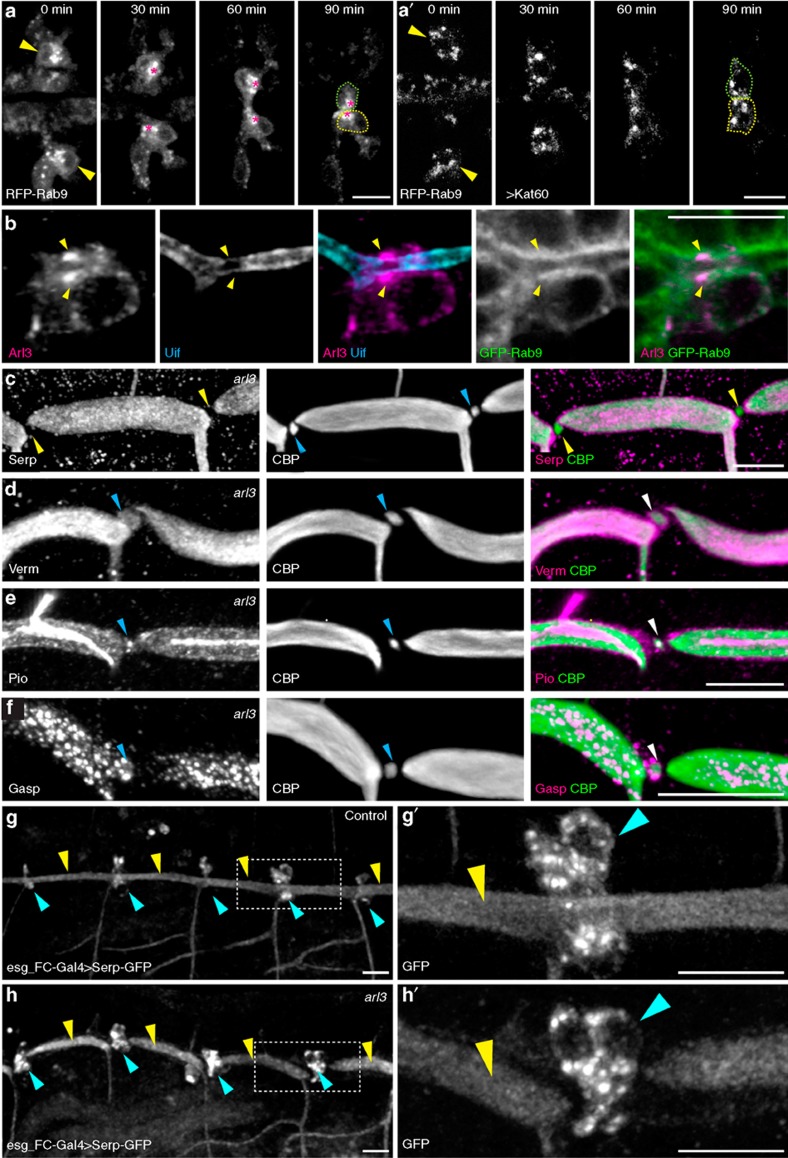
RFP-Rab9 vesicle trafficking in dorsal-branch FCs. (**a**) In dorsal-branch FCs (indicated by arrowheads and dotted outlines), RFP-Rab9 vesicle clusters (asterisks) were localized near the FC contact site. Anterior, left. (**a'**) Clustering of RFP-Rab9 vesicles was inhibited when microtubule was disrupted by Kat60. (**b**) Arl3 was also concentrated adjacent to the apical cell membrane (labelled with Uif) and co-localized with GFP-Rab9 at the FC contact site of the dorsal trunk (yellow arrowheads). (**c**–**f**) A specific secretion defect in *arl3*-mutant embryos: fusion points of stage-16 dorsal trunk were stained for CBP and Serp (**c**), Verm (**d**), Pio (**e**) or Gasp (**f**). Chitin, Verm, Pio and 2A12 were present in the lumen isolated from the FC contact site (blue arrowheads), whereas Serp (yellow arrowheads) was missing. (**g**,**h**) Test of directed Serp trafficking: Serp-GFP was expressed in FCs (blue arrowheads) using the *esg_FC-Gal4* driver in control (*arl3*^*1*^*/+*, **g**) and *arl3*^*1*^ mutant (**h**) embryos, respectively. Secreted Serp-GFP was present throughout the lumen in control embryos (yellow arrowheads in **g**) but absent from the FC contact site in *arl3*^*1*^ embryos (blue arrowheads in **h**). (**g'**, **h'**) Enlargement of the boxed regions in **g** and **h**, respectively. Scale bar, 10 μm.

Based on our observation that the Golgi apparatus moved towards the site of contact between FCs (Fig. 2g), we reasoned that the localization of secretory machineries is probably influenced by microtubule polarity. Rab9 is required for the endosome-to-Golgi trafficking of a key luminal component, the chitin deacetylase Serpentine (Serp)^33^,^34^,^35^. RFP-Rab9's localization in FCs was similar to that of the Golgi apparatus and of Arl3, another small GTPase that is essential for tracheal-branch fusion (Fig. 6a,b)^19^,^20^. We therefore examined the secretion of luminal components into the new lumen formed between FCs. Fusion points of dorsal trunk were observed for this study, because detailed analysis of lumen formation is possible with their large tube diameter. In *arl3* mutants, isolated chitin-filled lumen was found at the contact site of dorsal-trunk FCs (Fig. 6c) and the lumen contained secreted luminal proteins: the chitin deacetylase Verm^33^,^34^, the ZP-domain protein Pio^36^ and the chitin-binding protein Gasp^37^ (Fig. 6d–f). However, Serp was specifically missing in the isolated lumen (Fig. 6c). To determine whether FCs can secrete Serp, we expressed Serp-GFP by the FC driver and found that Serp-GFP was secreted and filled the entire tracheal lumen (Fig. 6g). In contrast, in an *arl3*-mutant background, Serp-GFP was absent at the FC contact site but filled the rest of the tracheal lumen (Fig. 6h). These results indicate that Arl3 is specifically required for the directed secretion of Serp into the lumen in the interface between paired FCs, but not for its secretion into FC–stalk cell interfaces.

### Rab9 overexpression overrides the requirement for Arl3

**Figure 7:**
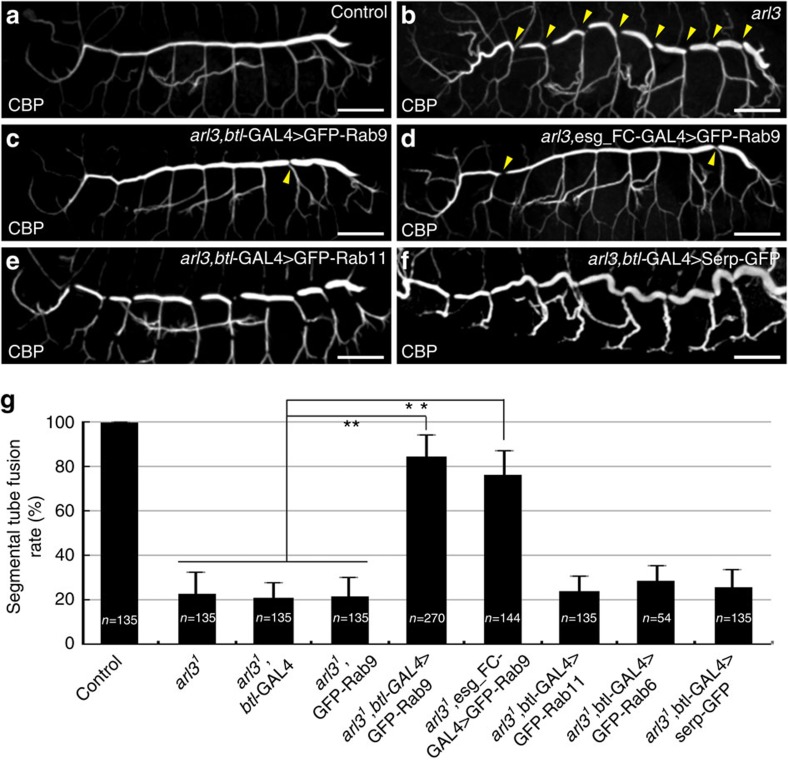
GFP-Rab9 overexpression rescued the *arl3*-mutant phenotype. (**a**,**b**) Control and *arl3*-mutant embryos were stained with CBP. Yellow arrowheads indicate a failure of branch fusion in the dorsal trunk of *arl3*-mutants. (**c**,**d**) GFP-Rab9 expressed specifically in the whole tracheal system by the *btl*-GAL4 driver or in FCs by the *esg_FC-Gal4* driver rescued the branch-fusion defect in *arl3* mutants. Yellow arrowheads indicate fusion-failure points remaining in the rescued embryos. GFP-Rab11 (**e**) and Serp-GFP (**f**) expression failed to rescue the *arl3* phenotype. (**g**) Quantification of the dorsal trunk fusion rate in different genotypes; 100% corresponds to nine successful fusion events in the ten tracheal metameres on one side of the embryo. Rescue activities of GFP-Rab9, GFP-Rab11, GFP-Rab6 and Serp-GFP were tested. Error bars indicate s.d. **The fusion rate was significantly higher in *arl3* mutants overexpressing GFP-Rab9 than in *arl3* mutants without GFP-Rab9 overexpression, by Student's *t*-test (*P*<0.01). Scale bar, 50 μm (**a**–**f**).

The aforementioned results suggested that Rab9-dependent Serp trafficking might be an important function of Arl3. To test this idea, we examined whether Rab9 overexpression could rescue the *arl3*-mutant phenotype. We found that GFP-Rab9 expressed by *btl-Gal4* or *esg_FC-Gal4* rescued the fusion defect of *arl3* mutants (Fig. 7a–d,g). However, GFP-fusion proteins of other small GTPases, Rab11 and Rab6, or of Serp failed to rescue the *arl3* phenotype (Figs. 7e–g and 6h). These results indicate that Rab9-dependent recycling and apical secretion is a crucial component of the Arl3-dependent conversion of paired FCs into a torus shape.

## Discussion

Previous studies showed that F-actin-enriched cell protrusions form in the tip of migrating tracheal branches^16^,^17^,^18^,^38^. Here we showed that tracheal FCs form polarized microtubule bundles oriented towards the leading edge of the migrating cells. The function of these microtubules is twofold: to concentrate E-cadherin to the newly contacted cell interface and to initiate the formation of new adherens junctions. We found that the E-cadherin that accumulated at the new cell interface is not recycled from the cell surface, but is instead drawn from a newly synthesized pool and recruited preferentially to the FC contact site, and not to existing adherens junctions between FCs and stalk cells. We speculate that the forward reorientation of the MTOC and the polarization of the microtubule plus ends towards the leading edge underlie the preferential deposition of E-cadherin at the FC contact site^39^. We considered one possible mechanism of preferential deposition, in which microtubules transport endosomes containing E-cadherin towards the contact site and we attempted to test this model by imaging vesicular trafficking of the complex containing E-cadherin-GFP and other adherens junction components. We did not find definitive evidence for this model. However, we observed that the Golgi apparatus shifted forward, towards the FC contact site, and that an E-cadherin-GFP signal increased in the plasma membrane before becoming concentrated at the contact site. Based on these observations, we favour a model in which the relocalization of the Golgi apparatus near the leading edge of the FC provides a source for E-cadherin that is deposited locally in the plasma membrane and the *trans* association of E-cadherin between the FCs nucleates a further concentration of E-cadherin via *cis* clustering^40^. Brodu and colleagues^15^,^41^ have shown that MTOC components are located apically in stalk cells and the microtubule function is required for the apical assembly of adherens junction proteins Par-3 and E-cadherin through regulation of recycling endosomes. This mechanism appears different from FCs, as assembly of new adherens junction occurs in the cell interface enriched with microtubule plus ends opposite to the centriole located in the proximal side.

A second microtubule function was discovered in this study, which was to equalize the contraction in FC pairs after contact. The coordinated contraction in FC pairs pulls two FC–stalk cell junctions simultaneously towards the FC contact site. The contractile force comes from a myosin-driven process; the microtubules may serve as a ‘ratchet' to fix the length of the FCs after each round of contraction. When microtubules were inhibited, the FCs relaxed to their original length after contracting, which delayed the overall fusion process. When microtubules were destabilized, branch fusion proceeded, albeit with delays and imbalances, and fusion was eventually completed. Even in this condition, the conversion of the FC cells into a torus shape occurred simultaneously, suggesting that there is a mechanism to coordinate the fusion event in FC pairs. The proposed ratchet-like function of microtubule must in some way be linked to the contractile activity of myosin. One good candidate molecule for coupling the actomyosin contraction to the microtubule function is Short-stop (Shot), which belongs to the conserved spectraplakin family of cytoskeletal proteins^42^, and was shown to be required for tracheal branch fusion^43^. The involvement of microtubules and Shot in the ratchet-like mechanisms observed in several contraction-dependent morphogenetic events^44^,^45^,^46^ should be tested in the future.

It is interesting to note that balancing of the force applied to the E-cadherin conjugated cell interface via microtubule plus ends is similar in configuration to the mitotic spindle, where microtubule plus ends attached to the kinetochore of each of paired sister chromosomes applies pulling force to each spindle pole^47^. The equal number of cadherin-catenin complex in each side of the FC interface associated with *trans*-conjugated E-cadherin pairs in the FC interface may provide a platform for microtubule plus end attachment for generating balanced contractile force.

When FCs are fully contracted, the plasma membrane of the two adherens junctions in each FC are connected in a single burst and cell pairs are converted simultaneously into a torus shape. This process requires Arl3 GTPase, which associates with the microtubules and intracellular vesicles concentrated at the FC contact site^19^,^20^. Here we showed that Arl3 is required for directed Serp trafficking, and that GFP-Rab9 overexpression overrides the requirement for Arl3. We propose that the microtubule-dependent transport of the Golgi apparatus and endosomes facilitates the concentration of Rab9 and Arl3 at the FC contact site, where they act together to increase the concentration of Serp in the lumen. The deacetylation of chitin converts it to the more hydrophilic form chitosan. The increase in water absorption by chitosan would cause the luminal matrix gel to swell, simultaneously pushing the plasma membranes of the FC interface closer to the plasma membrane of the FC–stalk cell interface so that the membrane-fusion machinery triggers the conversion of the paired FCs into a torus shape.

A number of issues remain to be explained. Lumen formation in FCs was clearly detected *arl3* mutants, but not in the normal context. This is probably because very small lumen is sufficient to trigger fusion of wild-type FCs. Although Arl3 is absolutely required for fusion, Rab9 and Serp are not, suggesting that the proposed luminal-matrix swelling due to chitin deacetylation is not the sole mechanism of plasma membrane fusion, and additional Arl3-regulated process of fusion control must exist. Moreover, additional Rab9 cargo that acts together with Serp to rescue the *arl3* mutants is predicted. To uncover the entire fusion process, it will be necessary to search for additional Arl3 and Rab9 targets, and to analyse FC-specific membrane trafficking and secretion.

## Methods

### *Drosophila* strains

The *esg_FC-Gal4* strains used here were constructed by injecting a plasmid containing a genomic fragment upstream of the *esg* gene into the pGaTB vector^48^ and by isolating insertions in the second and third chromosomes. The fusion-cell enhancer was identified during a study of *esg* regulatory elements^49^,^50^. The *esg_FC-Gal4* strain were constructed by inserting the 7,259 bp of genomic DNA (position 15313739–15320998 of NT_033779) upstream of *esg* into the P{CaSpeR-hs} vector (FBtp0000164), whereas YY, MT-M and SH were associated with the Genetic Resource Center, National Institute of Genetics in Mishima, Japan. This construct was described as *pAs-Gal4* expressed in the peripheral amnioserosa^51^. *UAS-p120ctn-tagRFP* was constructed with a p120ctn sequence amplified from the complementary DNA library^52^ and tagRFP (Evrogen) was constructed as described elsewhere^23^. *UAS-3xtagRFP* was constructed by cloning three copies of tagRFP as a tandem fusion. UAS-tagRFP-tau was constructed by fusing tau's microtubule-binding domain (amino acids 165–327) to the carboxy terminus of tagRFP. *UAS-TagRFP-Jupiter* was constructed by fusing Jupiter open reading frame spanning codon 2 to terminator sequence to the C-terminal end of Tag-RFP. *UAS-EB1-GFP* was constructed by Masako Kaido and Leo Tsuda. All of the fluorescent-protein constructs were cloned into pUAST vector and transformed into germ lines^48^,^53^.

Kat60's microtubule-severing activity was originally discovered in a screen of Gene Search strains^54^ for effectors interfering with tracheal morphogenesis (Kenzi Oshima, personal communication; P{w^+mC^=GSV6}GS10360 insertion).

The following fly strains were used: *btl-Gal4* (ref. 55), *UAS-GFP-moe*^56^, *UAS-Spas*^57^, *UAS-GFP-PACT*^27^, *UAS-DE-cad-GFP*^58^, *UAS-GFP-Zip* and *UAS-GFP-DN-Zip*^59^; the UAS-IR strain for DE-cad RNA interference (RNAi) (NIG Stock Center, 3327R-2), *UAS-tau-GFP*^60^, *UAS-Nod-lacZ*^26^, *UAS-GFPS65T* inserted in 2L (FBti0013280), UAS-Grasp65-GFP.2 (FBti0040816) and *UAS-Serp-GFP*^34^; *rab9*^*56*^, *rab9*^*199*^ and *UAS-TagRFP-Rab9* (ref. 35); and *arl3*^*1*^ and *UAS-mCherry-CAAX*^19^.

To compare the effect of Spas and Kat60 overexpression on the tracheal branch fusion, we imaged DB migration under condition of each effector expressed by *btl-Gal4*. DB migration was delayed by Kat60 and fusion failed frequently (31% *n*=26), whereas success rate was higher in Spas (85%, *n*=26) or control (100%, *n*=11).

### Staining

The following antibodies and dilution were used for staining: rabbit anti-GFP (Medical & Biological Laboratories Co., Ltd, #598, 500 × ), anti-beta-galactosidase (MP Biomedicals, #0855976, 1000 × ), rat anti-DE-cad (DCAD2, 20 × )^61^, mouse anti-Gasp (2A12, DSHB, 2 × ), anti-Serp (300 × ) and Verm (300 × )^34^, anti-Pio (50 × )^36^, anti-Arl3 (500 × )^19^ and CBP-Alexa 488 (50 × )^35^. Embryos were dechorionated with 1/2 diluted commercial bleach and were fixed by mixing vigorously in the mixture of 2 ml 4% paraformaldehyde in PBS and 2 ml heptane in liquid scintillation vial for 30 min at room temperature. After removal of heptane, embryos were devitelinized by addition of 2 ml methanol and vigorous mixing. Devitellinized embryos were washed two times with methanol and rehydrated with PBS. Embryos were stained as described previously^56^. Blocking, antibody incubation and washing were performed with 1% BSA and 0.2% Triton X-100 in PBS. The anti-Katanin 60 antibody (100 × ) was generously donated by Naoyuki Fuse and Fumio Matsuzaki.

### Microscopy

Time-lapse imaging of living embryos was performed with Olympus FV1000 with GaAsP detectors^19^. FRAP analyses were performed on tracheal cells expressing DE-cadherin-GFP using a confocal microscope equipped with a second scanner and a × 60 oil-immersion lens (FV-1000, Olympus). The sampling rate was every 10 s and specific locations were bleached with a 50-mW, 405-nm diode laser. EB1-GFP was imaged using a TCS SP5 II (Leica) equipped with a × 63 oil-immersion lens (numerical aperture=1.4). Optical sections (0.46 μm × 13) were scanned at 200 frames every 0.098 s. Infrared laser illumination was applied as a 1-s pulse at 60 mW and the embryos were imaged in 30-s intervals^25^.

### Measurement and data processing

All imaging data were processed and measured with applications developed in-house; these data were written in C and Objective-C and compiled as figures with ImageJ (Rasband, http://imagej.nih.gov/ij/). Measurements were processed with Ruby (http://www.ruby-lang.org/) and R (R Development Core Team, http://www.R-project.org/). The spatial coordinates (*x*–*y*–*z*–*t*) of areas with high signal intensity (E-cadherin-GFP, p120ctn-RFP or GFP-PACT) were tracked using software that searches for the local maximum within a three-dimensional volume manually specified by operators. Because of its concentrated localization, the E-cadherin fluorescence intensity at FC contact sites was estimated by taking the mean of the pixel intensities within a circle centred at the peak coordinate with a radius of 10 pixels (0.65 μm). The mobile fractions for FRAP analyses were estimated by fitting the fluorescence intensities to the following exponential equation:


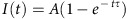


FC deformation was assessed by the shortening of the distance between the two FC–stalk cell interfaces (L–R) or between the FC contact site and the stalk-cell contact site (C–L and C–R). The particle image velocimetry analysis of EB1-GFP was performed on maximal *z*-projections of a confocal stack. In this analysis, 0.52-μm square windows were placed on the image for every 0.38 μm of *x*–*y* coordinates and the shift of the EB1-GFP signal in each window was estimated by searching within the next time point for the adjacent pixels by shifting original window up to 8 pixels along both *x* and *y* axes. The shift value showing the highest correlation of image pattern to the original window was recorded as vector. Tracks computed along 200 frames were mapped on a 25 × 25 grid of 0.38-μm squares and their direction and velocity were represented as bars.

### Numerical calculation of tug-of-war frequency

To compare the frequency of tug-of-war occurrence between wild-type and MT perturbed conditions, we measured the distance from FC contact site to FC/SC contact site (left and right) in every frames and calculated their change per time frame (displacement). Pairs of displacement value at each time point are then multiplied and the time points of which resulted in negative products were defined as ToW situation. Frequency of ToW situation was 72/154 (46.8%, 6 time series) and 112/180 (62.2%, 7 time series) for FC pairs in control and microtubule destabilized conditions, respectively. This result showed the frequencies of ToWs were significantly increased in MT-destabilized condition (Fisher's exact test, *P*=0.006).

To simulate ToWs *in silico*, 300 pairs of random numbers were generated from Gaussian distribution with sample mean and s.d. of experimentally measured FC deformations (−0.22±0.65 μm min^−1^ for control and −0.17±0.79 for spas). Probability distributions of simulated ToW frequencies mimicking both conditions were estimated by repeating this trial for 10,000 times.

### Quantifying the effect of E-cadherin RNAi in the trachea

UAS-*shg* RNAi constructs were expressed with *btl-Gal4* and embryos were fixed, stained with anti-E-cadherin and confocal image stacks were taken from stage-15 embryos. E-cadherin signal intensity in the epidermis and trachea (stalk cells of DB) was measured by setting region of interest with line tool (ten cell boundaries for each tissue per embryo), background subtracted and the intensity ratio of trachea and epidermis was calculated. Signal intensity ratio was 1.62±0.33 for control (*N*=6) and 1.18±0.14 for E-cad RNAi (*N*=4). This is ∼27% reduction of E-cadherin protein level (*P*=0.0397, Student's *t*-test, two tailed).

## Additional information

**How to cite this article:** Kato, K. *et al*. Microtubule-dependent balanced cell contraction and luminal-matrix modification accelerate epithelial tube fusion. *Nat. Commun.* 7:11141 doi: 10.1038/ncomms11141 (2016).

## Supplementary Material

Supplementary Figures Supplementary Figures 1-4

Supplementary Movie 1 Time course of dorsal branch migration and fusion and their relationship to the epidermis. GFP-moe was expressed in tracheal FCs and pAs by esg_FC-Gal4, and in the sqh-GFP-moe. Lower panel corresponds to a stacked x-z section in the boxed area of dorsal view in top panel. Scale bar in lower right is 10 μm and time stamp is shown in top-right. Original movie used to prepare Fig. 1c.

Supplementary Movie 2 Microtubule dynamics in tracheal FC and pAs revealed by tau-GFP marker expressed by the esg_FCGal4 driver. Original movie used to prepare Fig. 1d.

Supplementary Movie 3 Double labeling of tau-RFP (top, red) and GFP-moe (middle, green) driven by btl-Gal4. Related to Fig. 1e.

Supplementary Movie 4 High-speed imaging of E-cadherin trafficking during the first 3 minutes after contact of FCs. Related to Fig. 2f.

Supplementary Movie 5 Dynamics of myosin localization in DB fusion site revealed by esg_FC > GFP-Zip. Related to Fig. 3a.

Supplementary Movie 6 Laser perturbation of fusion cell contraction. Embryos carrying btl-Gal4, UAS-GFP-moe and UASp120ctn-RFP was illuminated with 1440 nm laser (60mW) for 1 second at one of fusion cells (left) of DB5 (t=0). The embryo was imaged with PlanApo 60x NA1.40 oil IR lens with 4x zoom. Then 512 x 512 pixel images of 1-mm-thick sections were captured every 30 sec for 20 minutes. Original movie used to prepare Fig. 3b.

Supplementary Movie 7 Effect of myosin inhibition on fusion of DB. GFP-DN-Zip was co-expressed with p120ctn-RFP by btlGal4. Top panel; x-y view, bottom panel: y-z view. Most of FCs reached the midline and accumulated p120ctn-RFP at the interface. But p120ctn-RFP was not concentrated into a spot and FC failed to contract. Original movie used to prepare Fig. 3d.

Supplementary Movie 8 Dynamics of microtubule plus end. FC and TC of btl > EB1-GFP embryo were imaged. The branch was directed toward 10 o'clock direction. Left: original movie. Right: overlay of PIV tracks. Original movie used to prepare Fig. 4c.

Supplementary Movie 9 Double labeling of EB1-GFP (green) and membrane marker (red) imaged in a btl > EB1-GFP, mRFPCAAX embryo. Dorsal branch was directed toward 2 o'clock direction. EB1 comet repeatedly entered into filopodia. Original movie used to prepare Fig. 4d. Scale bar: 10μm.

Supplementary Movie 10 Double labeling of tau-RFP (top, megenta) and E-cadherin-GFP (middle, green). Horizontal view of the focal plane covering FC. Related to Fig. 4e.

Supplementary Movie 11 Effect of Kat60 on microtubule stability visualized by tau-RFP imaging. Left: control, right: Kat60 expressed by btl-Gal4. Related to Fig. 4F.

Supplementary Movie 12 Effect of Kat60 on adherens junction formation. p120ctn-RFP expression in FCs was imaged in control (top) and Kat60 (bottom) expressing trachea. Central signal correspond to FC contact site. Frame rate 18 sec. Original movie used to prepare Fig. 4i.

Supplementary Movie 13 Microtubule inhibition caused unbalanced FC contraction. Control (top) and Spas expressing FCs are labeled with E-cadherin-GFP and tagRFP. esg_FC-Gal4 driver used here additionally labeled pAs. Each panel consists of x-y and x-z stacks of dorsal views of fusion event in one metamere. Vertical lines mark fusion cell adhesion site (L, C, R). Original movie used to prepare Fig. 5a.
