# Supplementary material for: Microtubule-dependent balanced cell contraction and luminal-matrix modification accelerate epithelial tube fusion
Source: Nat Commun. 2016 Apr 12;7:11141. doi: 10.1038/ncomms11141 (PMC4832058; doi:10.1038/ncomms11141)
Supplement: Supplementary Figures — 1-4 [file ncomms11141-s14.pdf]

Supplemental Figure 1

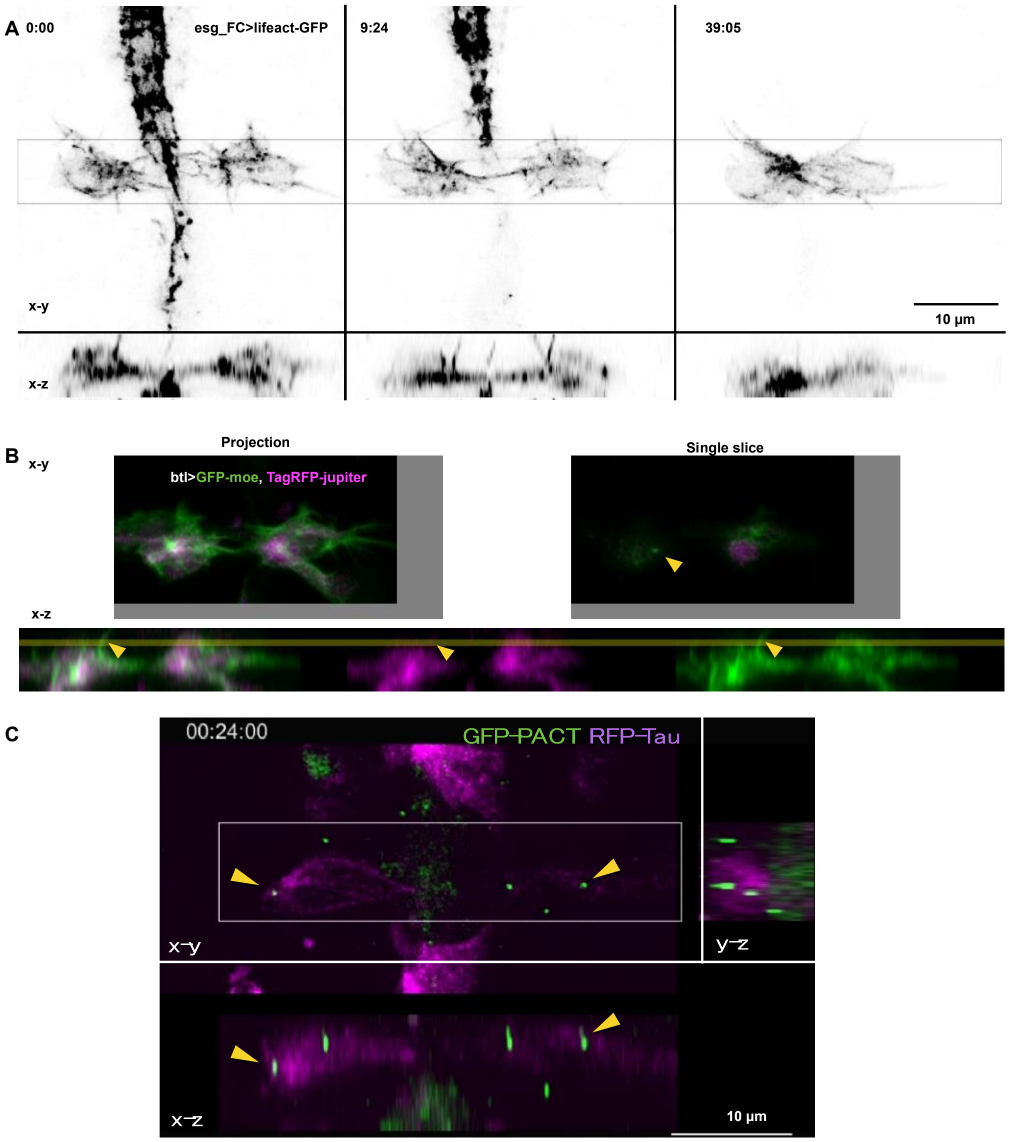

A. Migration of fusion cells in DB of a stage 16 embryo labeled with F-actin marker Lifeact-GFP driven by esg_FC-Gal4. Anterior, up. Vertical lines of signals in the center of 1:11 and 9:24 frames are pAs. Top row: x-y view. Bottom row: y-z view showing vertical filopodia.

B. Simultaneous labeling of microtubule with TagRFP-Jupitor and F-actin with GFP-moesin. x-y plane of projection (top left) and single slice (top right) across the plane indicated with yellow line in the bottom x-z section. Arrowhead indicates the

position of vertical filopodia.

C. Localization of the centriolar marker GFP-PACT in fusion cell. GFP-PACT expressed by ubiquitin promoter was imaged in esg_FC>RFP-Tau embryo. In the fusion cell in the left side, a dot of GFP-PACT was found in the center of fan-shaped microtubule arrays. Expression of RFP-Tau was low in the right side.

Supplemental Figure 2

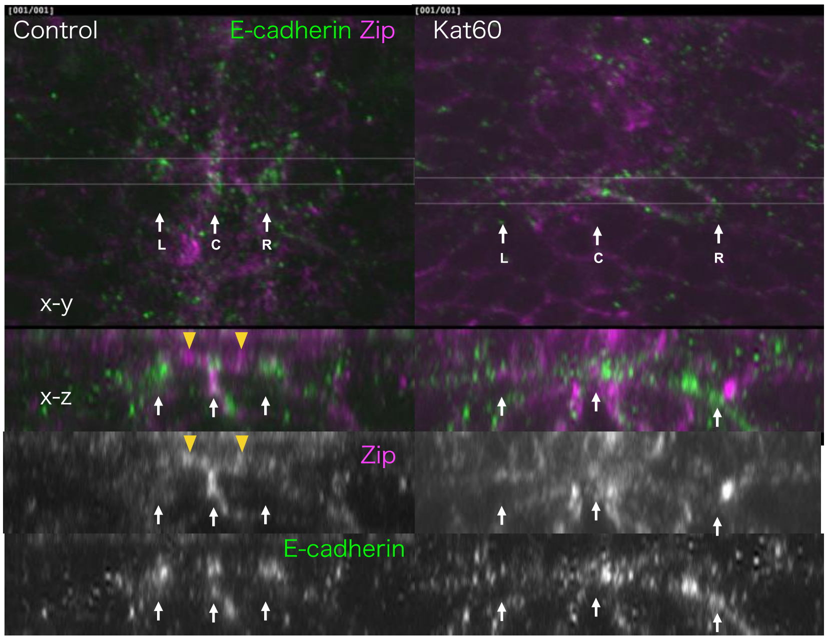

Myosin cable formation requires proper microtubule organization. btl-Gal4 or btl>Kat60 embryos were stained with anti E-cadherin (green) and anti Zip (magenta). FC contact sites (L, C, R) are marked with white arrow and Myosin cables in the control fusion cell was marked with yellow arrowhead. Such a structure was lost in Kat60 expressing cells.

Supplemental Figure 3

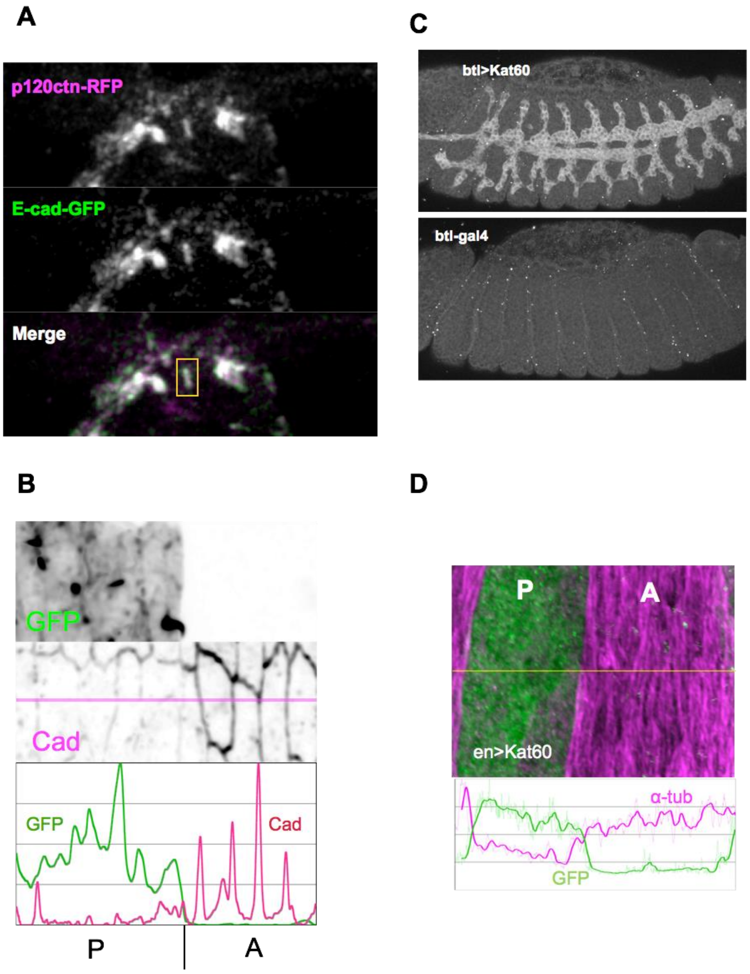

A. Colocalization of adherence junction marker p120ctn-RFP and E-cadherin-GFP. UAS construct of each marker was expressed by btl-Gal4 and imaged at the time of dorsal branch fusion in stage 16. FC contact site is boxed. B. Inhibition of E-cadherin expression by RNAi. Epidermal image of stage 15 embryo carrying en-Gal4, UAS-E-cadehrin RNAi and UAS-GFP-moe. E-cadherin antibody staining was greatly reduced in P compartment where en-Gal4 is active. C. Overexpression of endogenous *Katanin 60* gene was overexpressed by btl-Gal4 via activation of nearby insertion of gene search element P{GSV6} containing UAS and GFP (GS10360). High level of Kat60 expression was confirmed by antibody staining. Lower panel shows *btl-Gal4* embryo used as a negative control. D. Microtubule labeled by alpha-tubulin antibody was reduced by Kat60 expressed by *en-Gal4* in P compartment. GFP was expressed from the P{GSV6} element.

Supplemental Figure 4

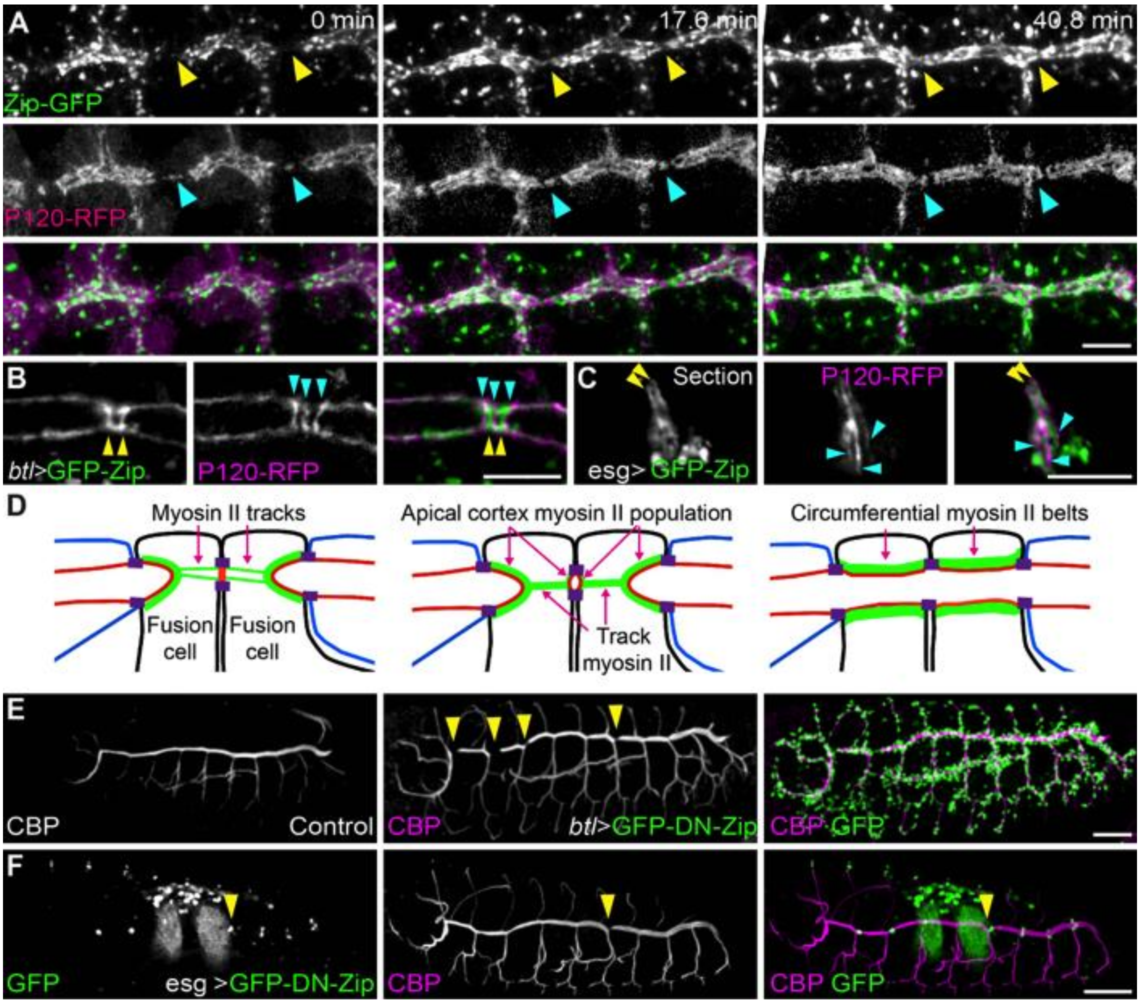

A. Time-lapse images of Zip (Non-muscle myosin II)-GFP and P120ctn-RFP expression during dorsal trunk fusion in stage 14 embryos. Blue and yellow arrowheads indicate P120ctn-RFP and GFP-Zip accumulation at the FC contact site, respectively. Cable of GFP–Zip appeared later than that of p120-RFP. B. Localization of GFP-Zip and P120ctn-RFP in the fusion point of stage 16 DT. Blue arrowheads show three P120ctn rings, separated by two wider Zip belts (yellow arrowheads). C. Similar localization pattern was observed by the expression of FC-specific expression of GFP-Zip and P120ctn-RFP. D. Schematic diagram of actomyosin belt formation in the fusion cells. E. Expression of dominant-negative Zip in all tracheal cells interfered branch fusion (arrowhead). F. Expression of GFP-DN-Zip in FCs. Relatively weak effect in this case may reflect later onset of expression by esg_FC-Gal4. Scale bars represent 10 μm for A-C and 50 μm for E-G.
